# Comparative transcriptome and physiological analyses unveil differential responses of *Panax ginseng* adventitious roots to iron *vs*. zinc deficiency

**DOI:** 10.3389/fpls.2026.1744224

**Published:** 2026-01-30

**Authors:** Siyu Zhang, Chenguang Wang, Yun Zhong, Zhuo Jin, Xiangguo Li, Songquan Wu

**Affiliations:** College of Agriculture, Yanbian University, Yanji, China

**Keywords:** adventitious roots, ginsenosides, iron deficiency stress, *Panax ginseng*, transcriptome, zinc deficiency stress

## Abstract

Trace elements iron (Fe) and zinc (Zn) are essential nutrients for plant growth and development, and their deficiency significantly inhibits the physiological metabolism and bioactive compound accumulation of medicinal plants. As a high-value medicinal plant, *Panax ginseng* C.A. Mey. is susceptible to mineral nutrient stress, affecting its quality and yield. However, current research on the genetic regulatory mechanisms of Fe and Zn is relatively scarce. This study conducted a fundamental investigation by comparing the morphology, physiological metabolism, and transcriptome of P. ginseng adventitious roots under Fe and Zn deficiencies. Results showed that Zn deficiency more strongly inhibited biomass accumulation than Fe deficiency. Zn deficiency significantly reduced total ginsenoside content, whereas Fe deficiency specifically decreased protopanaxadiol-type ginsenosides. Transcriptome analysis identified 2388 and 1055 differentially expressed genes under Fe and Zn deficiency, respectively. Both deficiencies led to widespread downregulation of key ginsenoside biosynthetic genes, though Fe deficiency upregulated protopanaxadiol synthase (PPDS) and protopanaxatriol type ginsenosides (PPTS) expression, consistent with ginsenoside profiles. Iron and zinc deficiencies elicited distinct hormone-response patterns, characterized by broad alterations in auxin-related genes: iron deficiency suppressed jasmonic acid signaling but activated abscisic acid, whereas zinc deficiency suppressed abscisic acid. Additionally, cytokinin homeostasis was disrupted under both deficiencies, whereas ethylene signaling was enhanced, particularly under Fe stress. Fe deficiency triggered a typical Strategy I response, whereas Zn deficiency altered ZIP transporter expression. These findings reveal the specific expression changes of genes related to ginsenoside biosynthesis, hormone metabolic pathways, and Fe/Zn transport and homeostasis regulation in ginseng under iron deficient and zinc deficient stress conditions, laying a foundation for further in-depth research into the molecular response mechanisms of ginseng adventitious roots to iron and zinc deficiency during their growth and development.

## 1 Introduction

*Panax ginseng* C.A.Mey, a perennial plant in the Araliaceae family, is a cornerstone of traditional medicine. Ginsenosides (GDs) are the primary secondary metabolites of ginseng. They are glycosylated triterpenoid saponins that exhibit a range of effects, including immune regulation, antioxidant activity, and anticancer activity (Kim et al., 2017; Tao et al., 2023). According to the different glycosidic bonds, they are divided into protopanaxadiol type ginsenosides, protopanaxatriol type ginsenosides, and oleanolic acid type ginsenosides. PPD and PPT type ginsenosides are dammarane-type tetracyclic triterpenoids composed of isoprene units, accounting for 80% of the total ginsenosides (Chopra et al., 2023).

The biosynthetic pathway of ginsenosides is generally divided into three stages (Christensen, 2008), which may be differentially regulated by nutrient deficiency or excess. In the initial stage, the cytosolic mevalonate (MVA) pathway is the primary route for producing isopentenyl pyrophosphate (IPP) and dimethylallyl pyrophosphate (DMAPP) from acetyl-CoA. In contrast, the plastidial methylerythritol phosphate (MEP) pathway plays a complementary role (Zhao et al., 2014; Xu et al., 2017). The second stage: the key precursor, 2,3-oxidosqualene, was synthesized from IPP and DMAPP, catalyzed by various enzymes. The third stage catalyzes the cyclization of 2,3-oxidosqualene to form a dammarane skeleton (Huang et al., 2023). Subsequently, two key cytochrome P450 enzymes, protopanaxadiol synthase (PPDS) and protopanaxatriol synthase (PPTS), catalyze the formation of PPD-type and PPT-type aglycones, respectively (Liu et al., 2023). (**Supplementary Figure S1**) Then, glycosylation was performed at a specific position (PPD was C3/C20, PPT was C6/C20) to mediate the biosynthesis of the final ginsenosides (PPD-type and PPT-type saponins) (Han et al., 2011; Thimmappa et al., 2014). The biosynthetic pathway of ginsenosides is clear. However, the molecular mechanisms by which nutrient deficiency or excess influences this pathway remain to be elucidated.

Adventitious-root culture constitutes a well-established platform for large-scale ginsenoside production that circumvents the constraints of field cultivation while offering genetic stability and a shortened growth cycle (Xu et al., 2023). The nutrient composition of the culture medium directly governs ginsenoside biosynthesis; indeed, the availability of micronutrients such as iron and zinc has been shown to redirect secondary-metabolic flux in plant *in vitro* cultures (Liang et al., 2019). Iron and zinc deficiencies trigger distinct physiological and metabolic responses in plants. Iron deficiency leads to leaf chlorosis, reduced photosynthesis, and enhanced root organic acid secretion (e.g., citrate, malate) to mobilize iron (Rout and Sahoo, 2015; Stanton et al., 2022). Metabolically, it upregulates secondary pathways, such as coumarin and flavonoid biosynthesis, while suppressing primary metabolism (Rellán-Álvarez et al., 2011). Zinc deficiency causes growth stunting, chloroplast damage, and oxidative stress, activating antioxidant enzymes and promoting secondary metabolite accumulation (e.g., alkaloids, flavonoids) (Wang et al., 2016). Both stressors divert resources from growth to defense through distinct mechanisms. Despite the abundance of iron (Fe) in soil, its low bioavailability limits plant acquisition, rendering Fe deficiency a major agricultural constraint (López-Millán et al., 2013). As an essential cofactor in numerous enzymes, Fe is involved in critical processes such as nitrogen assimilation, respiration, and hormone biosynthesis. Its redox activity between ferrous (Fe²^+^) and ferric (Fe³^+^) forms underpins its role as a catalytic center in oxidoreductases. Aberrant Fe availability impairs cell division, secondary metabolism, and overall plant physiology (Kobayashi and Nishizawa, 2012).

Under iron deficiency, non-graminaceous plants, such as Arabidopsis thaliana, initiate the classic Strategy I response mechanism. This mechanism first improves the solubility and reduction efficiency of iron in the rhizosphere by means of root acidification, activation of iron reductase (ferric reductase oxidase 2) FRO2, and secretion of phenolic substances. It then completes iron absorption via the high-affinity transporter IRT1 (Iron-regulated transporter 1). Iron homeostasis is also regulated by transporters such as Natural Resistance–Associated Macrophage Protein 1 (NRAMP1) and vacuolar iron transporter 1 (VIT1) (Kim et al., 2006). This adaptive response is coordinated and regulated by bHLH transcription factors, of which (FER-like iron deficiency–induced transcription factor) FIT is particularly critical, which activates the expression of FRO2 and IRT1 (Zhang et al., 2023). To maintain iron homeostasis, the E3 ubiquitin ligase BRUTUS (BTS) acts as a key negative regulator. It prevents hyperactivation of the iron-deficiency response pathway by targeting and degrading the positive regulatory transcription factors involved in this process (Hindt et al., 2017). Notably, the transcriptional level of BTS itself is also strongly induced under iron-deficient conditions (Rodríguez-Celma et al., 2019).

Zinc (Zn²^+^) is an essential micronutrient used by ~9% of eukaryotic proteins, making it the second most abundant biological metal cofactor after iron (Stanton et al., 2022). In perennial ginseng, which has a high demand for zinc, zinc-dependent enzymes play a crucial role in maintaining membrane integrity, regulating auxin levels, controlling carbohydrate/protein/lipid metabolism, and regulating gene expression (Gupta et al., 2016; Singh et al., 2016). Notably, zinc constitutes the structural core of zinc finger proteins, which are critical for transcriptional regulation and plant development (Zhou et al., 2013). Zn transporters and transcription factors involved in zinc deficiency response and zinc homeostasis in plant cells, including ZRT-/IRT-like protein (ZIP) transporters, which facilitate zinc and iron movement. Under zinc-deficient conditions, certain ZIP family members, such as ZIP1 and ZIP2, are upregulated to enhance zinc absorption (Assunção et al., 2013). Other contributors include NRAMP proteins, Heavy Metal ATPases (HMA), Yellow-Stripe-Like (YSL) transporters, and Cation Diffusion Facilitators (CDF). Nicotinamide (NA), synthesized by nicotinamide synthase (NAS), serves as a key metal chelator, and Vacuolar Iron Transporter (VIT) proteins mediate vacuolar Zn storage (Chen S. et al., 2019). Expression adjustments in these transporters enable plants to adapt to Zn variability and sustain normal growth (Talke et al., 2006; van de Mortel et al., 2006).

Therefore, this study analyzed the physiological mechanisms of ginseng in response to iron and zinc stress and the expression of related genes by measuring physiological indices in ginseng adventitious roots under these stresses. This study investigated key genes in the ginsenoside biosynthesis pathway under nutrient stress to elucidate mechanisms of adaptation. The findings provide a foundation for future research on the molecular responses of ginseng adventitious roots to iron and zinc deficiency during growth and development.

## 2 Materials and methods

### 2.1 Plant materials and stress treatments

Experimental materials were Panax ginseng C.A. Mey. adventitious roots, induced and maintained through long-term subculture in our laboratory. Callus cells were induced from P. ginseng roots using the method described by Jiang et al (Jiang et al., 2016). Fresh ginseng roots were surface-sterilized with 2% (w/v) sodium hypochlorite (20 min, continuous agitation) and rinsed 3× with sterile distilled water. Disinfected root explants were inoculated onto MS medium (1 mg·L 2,4-D, 1% sucrose, 0.8% agar) in 90 mm Petri dishes, and cultured in the dark at 25 ± 2°C for callus induction. Adventitious roots regenerated from calli were transferred to liquid culture. Uniform root segments (approximately 1 cm in length) were aseptically inoculated into 250-mL Erlenmeyer flasks, each containing 75 mL of Murashige and Skoog (MS) basal medium (Sigma-Aldrich, USA) supplemented with 5.0 mg·L^-^¹ indole-3-butyric acid (IBA) and 30 g·L^-^¹ sucrose (pH 5.8 ± 0.2). The cultures were maintained in complete darkness at 25 C under continuous agitation at 100 rpm, with subculturing performed every 35 days.

Three experimental groups were established, each with three biological replicates (n=3). For each replicate, approximately one cm-long adventitious root segments were inoculated into three independent 250 mL conical flasks to form root clusters. After the growth period, the root clusters were harvested, pruned again into fragments approximately 1 cm, and pooled within each replicate to form uniform samples for stress treatment. Iron-deficient group (-Fe): Fe-EDTA omitted (0 mmol·L^-^¹ Fe²^+^); Zinc-deficient group (-Zn): ZnSO_4_·7H_2_O omitted (0 mmol·L^-^¹ Zn²^+^); Control group (CK): Standard MS medium containing 0.1 mmol·L^-^¹ Fe²^+^ and 0.15 mmol·L^-^¹ Zn²^+^. After 35 days of culture, adventitious roots from each replicate were harvested, immediately frozen in liquid nitrogen, and stored at -80 °C until RNA extraction. This experimental design yielded nine samples (3 treatment groups × three biological replicates), ensuring sufficient statistical power for transcriptome sequencing analysis.

### 2.2 Physiological assessment

Adventitious roots were washed with deionized water, and surface moisture was removed before fresh weight was measured. Following this, the samples were dried in an oven at 55°C until a constant weight was achieved (48 hours), and the dry weight was recorded.

Fresh tissue (1 g) was homogenized in 50 mM phosphate buffer (pH 7.8) containing 1% polyvinylpyrrolidone at 4°C. The homogenate was centrifuged at 12,000 × g for 15 min at 4°C, and the supernatant was used for enzyme assays: catalase (CAT) activity was determined by potassium permanganate titration, measuring H_2_O_2_ decomposition at 240 nm. Results were expressed as mg H_2_O_2_ decomposed per min per g fresh weight (FW). Peroxidase (POD) activity was assayed by measuring guaiacol oxidation at 470 nm, with one unit (U) defined as a 0.01 change in absorbance per min per g FW. Superoxide dismutase (SOD) activity was evaluated by measuring the inhibition of nitroblue tetrazolium (NBT) photoreduction at 560 nm. One unit corresponded to 50% inhibition of NBT reduction per g FW (Zhou et al., 2017; Xie et al., 2025).

For the saponin extraction and purification, we used the dried samples, which were ground into a fine powder (0.3 g). Each sample was extracted with 25 mL of methanol by refluxing at 75°C for 3 hours in a water bath. After cooling to room temperature, the supernatant was collected and evaporated to dryness using a rotary evaporator at 60°C. The residue was dissolved in 5 mL of chromatographic-grade methanol, then partitioned with 25 mL of ether (vortexed for 45 seconds, followed by phase separation for 40 minutes). The lower layer was subjected to four sequential extractions with water-saturated n-butanol (12,000 rpm, 5 min each). The combined n-butanol phase was concentrated and reconstituted in methanol (5 mL per 0.3 g sample), then filtered through a 0.45 μm membrane.

### 2.3 Liquid chromatography conditions

Liquid chromatography analysis was performed according to the method described by Fuzzati (2004). Separation was performed on a reversed-phase C18 column (Acchrom, five μm, 4.6 × 250 mm) maintained at 25°C. The mobile phase consisted of ultrapure water (phase A) and acetonitrile (phase B) delivered at a flow rate of 1.0 mL/min^-^¹. The column temperature was maintained at 25°C, the injection volume was 10 μL, and the detection wavelength was 203 nm. The gradient elution schedule was as follows: 0–13 min with A: B = 77:23; 13–33 min with 54:46; 33–45 min with 32:68; 45–60 min with 0:100; and 60–66 min with 77:23. Detection was conducted using an HPLC-UV detector (G1316A, Agilent Technologies, Germany) at 203 nm. The injection volume was 10 μL. In this study, Rb1, Rb2, Rc, and Rd were classified as ppd-type ginsenosides; rg1, Re, and Rf were divided into ppt-type ginsenosides.

### 2.4 RNA sequencing

RNA-seq library preparation and sequencing were performed by Biomarker Technologies Co., Ltd (Beijing, China). RNA concentration and purity were measured using a NanoDrop 2000 (Thermo Fisher Scientific, Wilmington, DE). RNA integrity was assessed using the RNA Nano 6000 Assay Kit of the Agilent Bioanalyzer 2100 system (Agilent Technologies, CA, USA). A total of 1 μg of RNA per sample was used as input for RNA sample preparation. Sequencing libraries were generated using the Hieff NGS Ultima Dual-mode mRNA Library Prep Kit for Illumina (Yeasen Biotechnology (Shanghai) Co., Ltd.) according to the manufacturer’s recommendations, and index codes were added to assign sequences to each sample. Raw data (raw reads) in fastq format were first processed using in-house Perl scripts. In this step, clean data (clean reads) were obtained by removing adapter- and poly-N-containing, as well as low-quality, reads from the raw data. At the same time, Q20, Q30, GC-content, and sequence duplication level of the clean data were calculated. All downstream analyses were based on high-quality, clean data (**Supplementary Table S7**). Clean reads were aligned to the Panax ginseng IPGA v1.1 reference genome (obtained from http://ginsengdb.snu.ac.kr/ (Jayakodi et al., 2018); total predicted genes: 59,352) using Hisat2 (2.0.4) tools (Zhao et al., 2018), achieving alignment rates of 94.78–96.65% (**Supplementary Table S8**). Differential gene expression analysis was conducted using DESeq2 on biological triplicates. Genes with |FC| > 1.5 and adjusted P < 0.05 (Benjamini–Hochberg method) were considered differentially expressed. All sequencing data are available in the SRA database under accession PRJNA1291973 (https://www.ncbi.nlm.nih.gov/sra/PRJNA1291973, accessed September 17, 2025). The results of the principal component analysis (PCA) are provided in **Supplementary Figure S3**.

### 2.5 GO classification and KEGG classification analysis

Gene Ontology (GO) enrichment analysis of the differentially expressed genes (DEGs) was performed using the clusterProfiler package based on the Wallenius non-central hypergeometric distribution (Young et al., 2010). Enrichment results were visualized in a bubble diagram, with the x-axis indicating gene ratios and the y-axis listing GO terms. Point size corresponds to the number of DEGs per term, and color represents the −log_10_(q-value). (http://www.geneontology.org/, accessed on 20 May 2025). Pathway annotation analysis was performed using the Kyoto Encyclopedia of Genes and Genomes (KEGG) mapping methodology (Kanehisa et al., 2004) (http://www.genome.ad.jp/kegg/kegg2.html, accessed April 20, 2025). We used the KOBAS (Mao et al., 2005) database and the clusterProfiler software to test the statistical enrichment of differentially expressed genes in KEGG pathways. The enrichment results are presented in a bubble diagram, with the y-axis indicating pathway names and the x-axis representing the enrichment factor. The size of each circle corresponds to the number of DEGs mapped to the pathway, and the color reflects the -log_10_(q-value).

### 2.6 Quantitative real-time polymerase chain reaction validation

To evaluate the reliability of RNA sequencing data and validate DEG expression patterns, 10 genes with distinct expression profiles were selected and validated by qRT-PCR. Gene-specific primers were designed using Primer Premier 5.1 software; sequences are provided in **Supplementary Table S9**. The 18S rRNA gene was used as an internal control. The Total RNA Pure kit (Beijing Zhuangmeng International Biological Gene Technology Co., Ltd.) was used to extract total RNA from the samples. After extraction, residual genomic DNA was removed by RNase-free DNase treatment, and RNA concentration and purity were determined using an ultraviolet spectrophotometer (Merinton, USA). Subsequently, RNA was reverse transcribed into cDNA using the Chuang Meng Biological Reverse Transcription Kit. Quantitative real-time PCR (qRT-PCR) was performed using the QuantStudio Real-Time PCR System (Thermo Fisher Scientific, USA) with the 2× SYBR Green qPCR Master Mix (Beijing Chuangjian Biotechnology Co., Ltd., China). Each 10 μL reaction contained five μL of Perfect Start Green qPCR Super Mix, 0.2 μL each of forward and reverse primers (10 μM), 100 ng of cDNA template, and nuclease-free water, for a final volume of 10 μL. The thermal cycling conditions were as follows: 10 min at 95°C, followed by 40 cycles of 95°C for 10 s, 60°C for 10 s, and 72°C for 20 s. Relative gene expression levels were calculated using the 2^–ΔΔCt method. All experiments were performed with three independent biological replicates, and data are presented as mean ± standard error (SE).

### 2.7 Statistical analysis

Biomass, ginsenoside content, antioxidant enzyme activity, and gene expression data were expressed as mean ± standard deviation. The significant difference between the two groups’ mean values was evaluated using a t-test (P < 0.05). The significant differences among multiple groups were assessed using analysis of variance (ANOVA) (P<0.05), supplemented by Duncan’s multiple-range test for *post hoc* pairwise comparisons (P<0.05).

## 3 Results

### 3.1 Physiological changes of ginseng under iron and zinc deficiency

**Figure 1:**
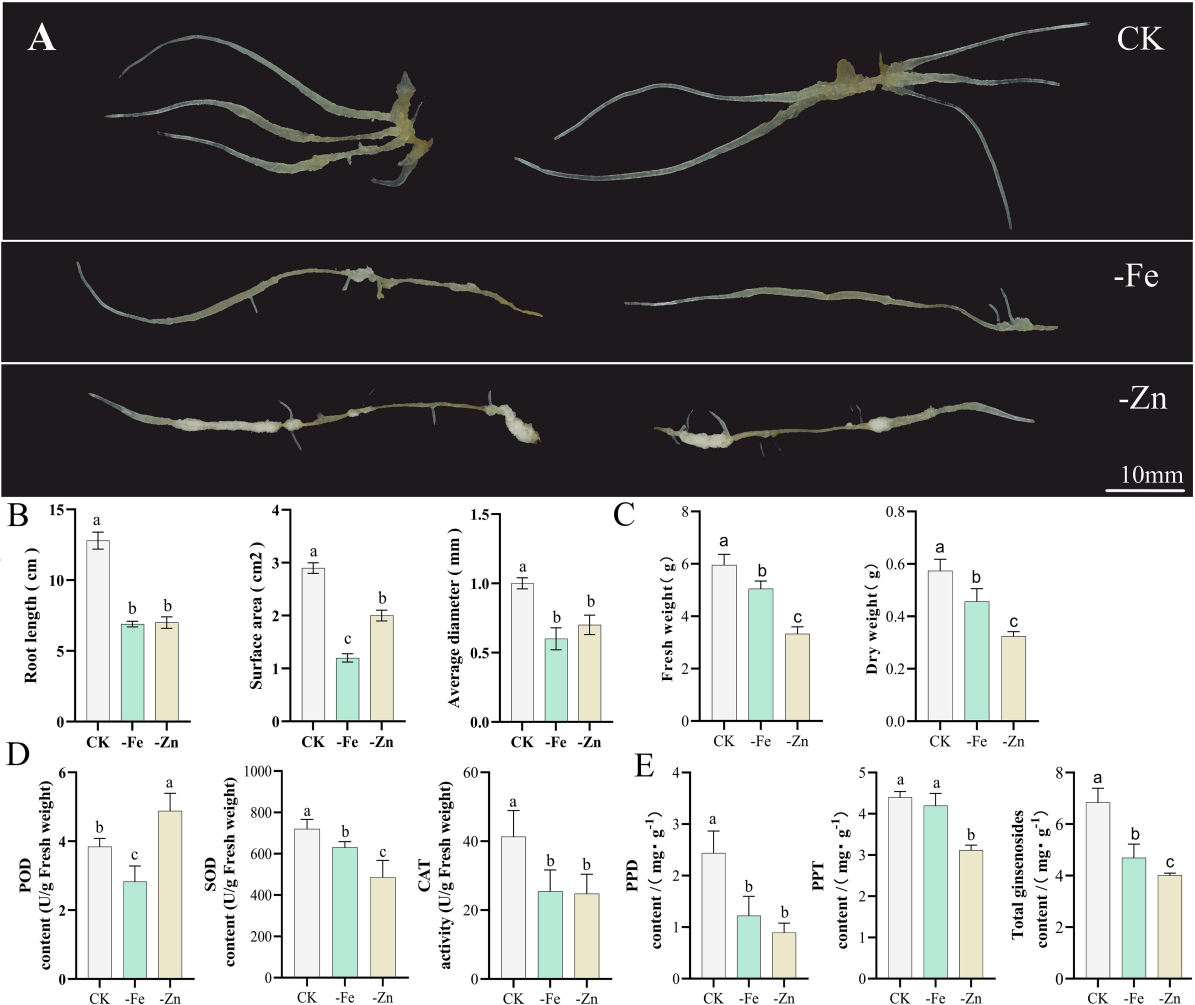
Phenotypic, physiological, and metabolic responses of *Panax ginseng* adventitious roots to -Fe and -Zn. **(A)** Morphological response of ginseng to nutrient deficiencies; **(B)** Root length, surface area, and average diameter of ginseng adventitious root; **(C)** Fresh weight and dry weight of ginseng adventitious root; **(D)** Changes in physiological characteristics of ginseng; **(E)** Ginsenoside content. The statistical significance between the data is expressed by different letters a–c, where different letters indicate significant differences at p < 0.05, and the error bars indicate standard deviations (SDs).

Under conditions of iron deficiency (-Fe) and zinc deficiency (-Zn), adventitious roots of ginseng exhibited distinct morphological alterations compared to the control (CK) in rotary shake flask cultures (**Figure 1A**). The roots of CK grew vigorously; the main roots were thick and strong, and the color was healthy light yellow. The root architecture of Fe was simplified, the main root became thinner and shorter, and the root tip became darker. The inhibition caused by -Zn was severe; the main root was thin and atrophic, and local swelling indicated a morphological abnormality caused by stress. The color was white. Quantitative assessment of root growth parameters—including root length, surface area, and average diameter—revealed that both deficiency treatments impaired root development, with the most pronounced growth and differentiation occurring in the control (CK; **Figure 1B**).

As shown in **Figure 1C**, zinc deficiency stress had a more pronounced adverse effect on ginseng biomass. The fresh weights under zinc deficiency were 3.33 g (44.03% lower than the control at 5.95 g), compared to 5.05 g (15.13% decrease) under iron deficiency. Similarly, dry weight declined by 43.86% (0.32 g *vs*. 0.57 g control) under zinc deficiency, compared with a 21.05% reduction (0.45 g) under iron deficiency.

By measuring the activity of antioxidant enzymes under iron deficiency and zinc deficiency, the physiological basis of adventitious root stress was further discussed. Under iron deficiency, the activities of POD, SOD, and CAT were consistently reduced. In zinc deficiency, SOD and CAT activities were also significantly suppressed, whereas POD activity was markedly elevated (**Figure 1D**).

Both iron and zinc deficiency also adversely affected ginsenoside biosynthesis. The total ginsenoside content declined from 6.83 mg·g^-^¹ in CK to 4.68 mg·g^-^¹ under -Fe and 4.00 mg·g^-^¹ under -Zn, representing reductions of 31.48% and 41.43%, respectively. Specifically, the content of PPD-type saponins was significantly reduced by iron deficiency, whereas PPT-type saponins remained unaffected. In contrast, zinc deficiency led to a significant decrease in both PPD and PPT saponin classes (**Figure 1E**).

### 3.2 Gene expression of ginseng under iron and zinc deficiency

**Figure 2:**
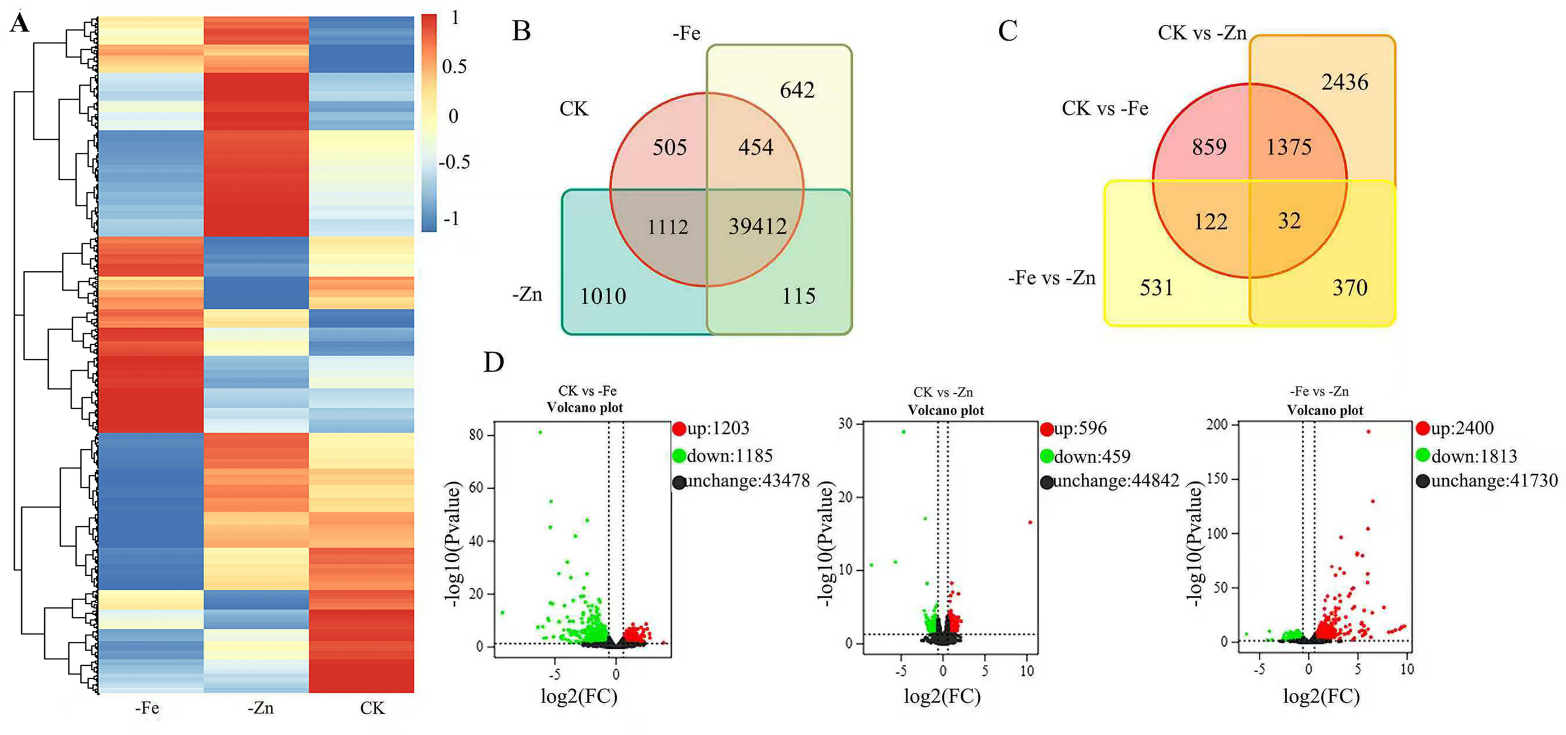
Transcriptomic profiling of Panax ginseng adventitious roots under -Fe and -Zn. **(A)** Heatmap of gene expression levels in ginseng adventitious roots. **(B)** A Venn diagram illustrating the common and unique number of gene expression under iron and zinc deficiency stress. **(C)** Volcano map presenting the DEGs of ginseng adventitious roots under iron and zinc deficiency stress. **(D)** Venn diagram of DEGs of ginseng adventitious roots under iron and zinc deficiency stress. CK, -Fe and-Zn represented control, iron deficiency stress, and zinc deficiency stress, respectively.

To investigate the global transcriptomic responses of ginseng adventitious roots to iron and zinc deficiency, we performed RNA-seq analysis under three culture conditions: CK, -Fe, and -Zn. A total of 50,814, 51,416, and 51,345 genes were expressed in the CK, -Fe, and -Zn samples, respectively. The overall gene expression patterns across samples are visualized in a heatmap (**Figure 2A**). A Venn diagram was constructed to illustrate the overlap and uniqueness of expressed genes among the three groups, revealing both adventitious root-specific and genotype-specific expression profiles under Fe and Zn deficiency (**Figure 2B**).

Differential gene expression analysis identified significant changes in response to each nutrient stress. In the Zn-deficient group, 1,055 DEGs were detected when compared to the control, comprising 596 upregulated and 459 downregulated genes (**Figure 2C**). Under Fe deficiency, a larger set of 2,388 DEGs was identified, comprising 1,203 upregulated and 1,185 downregulated genes (**Figure 2C**). Notably, a direct comparison between -Fe and -Zn treatments revealed an even greater number of DEGs, underscoring the distinct transcriptional reprogramming triggered by each specific nutrient deficiency (**Figure 2C**). A Venn diagram comparing DEGs across conditions further illustrates the shared and unique transcriptional responses to -Fe and -Zn stress (**Figure 2D**).

### 3.3 Gene Ontology and Kyoto Encyclopedia of Genes and Genomes enrichment analysis following iron deficiency and zinc deficiency stress in ginseng

We conducted Gene Ontology (GO) and KEGG pathway enrichment analyses to characterize the functional profiles of DEGs in ginseng adventitious roots under iron deficiency (-Fe) and zinc deficiency (-Zn) stress.

**Figure 3:**
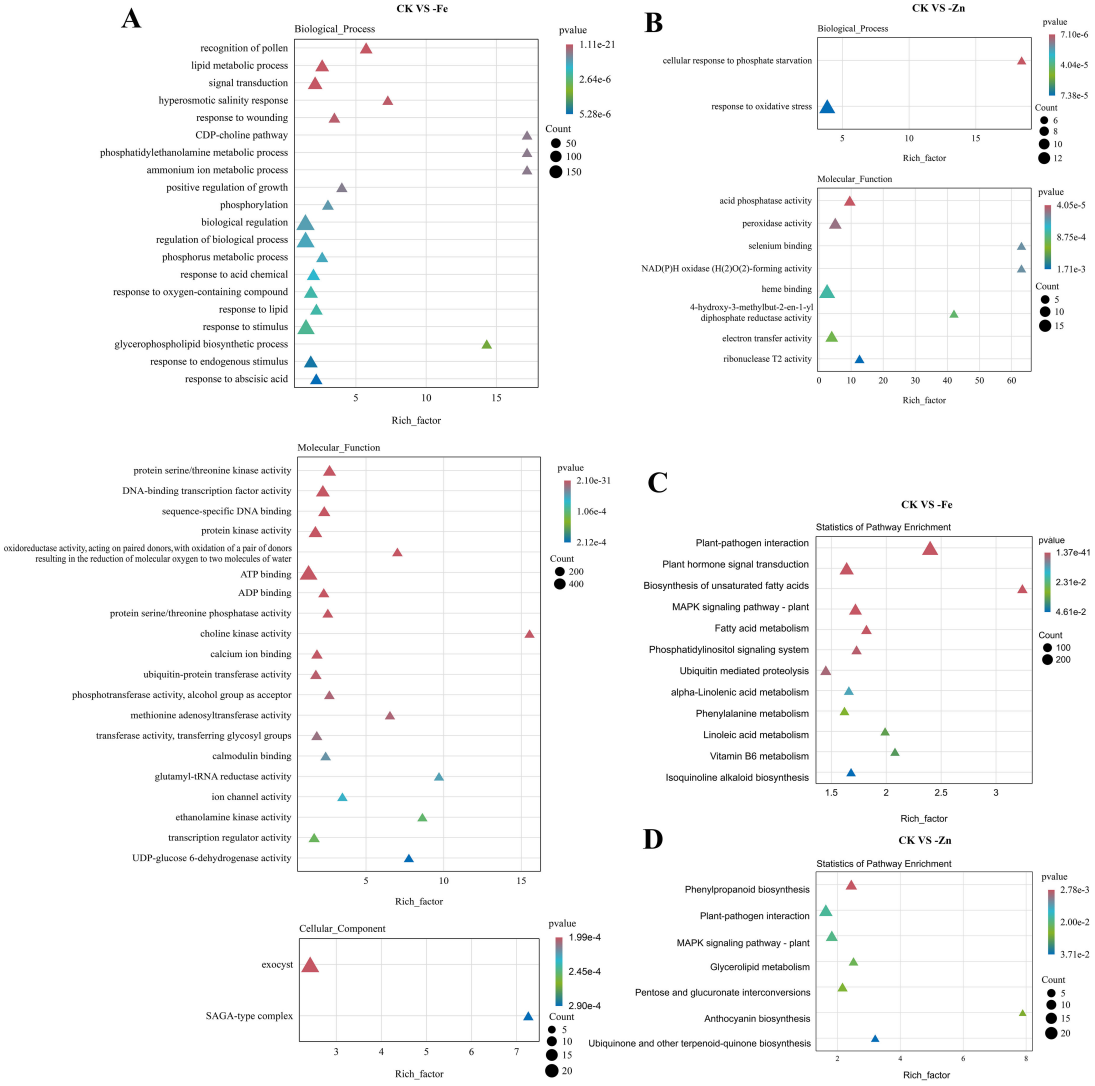
Enrichment analysis of up-regulated DEGs. **(A)** GO enrichment under Fe deficiency. **(B)** GO enrichment under Zn deficiency. **(C)** KEGG pathway enrichment under Fe deficiency. **(D)** KEGG pathway enrichment under Zn deficiency. All plots show terms/pathways with significant enrichment (adjusted P−value < 0.05). CK, –Fe, and –Zn represent control, iron deficiency, and zinc deficiency, respectively.

In the GO analysis, DEGs from the “CK *vs* -Fe” and “CK *vs* -Zn” comparisons exhibited consistent distributions across major GO categories (**Supplementary Figure S1**).To elucidate the differential responses to iron (Fe) and zinc (Zn) deficiency, we performed separate GO and KEGG pathway enrichment analyses on up- and down-regulated genes under each stress condition (**Figure 3**, **Supplementary Figure S4**, **Supplementary Tables S1**, **S2**). Combined GO functional analysis and KEGG pathway enrichment analysis of upregulated genes revealed that plants employ distinct molecular regulatory strategies in response to Fe- and Zn-deficiency stress. Under iron deficiency, significant enrichment was observed in biological processes (BP) related to cell proliferation regulation, DNA replication, and lipid metabolism. In contrast, molecular functions (MF) were primarily enriched for protein kinase activity and DNA-binding activity (**Figure 3A**). The most prominently enriched KEGG pathway was “plant–pathogen interaction,” suggesting that Fe deficiency triggers a robust immune-like response in plants. This was accompanied by the co-activation of multiple lipid metabolic pathways, such as unsaturated fatty acid biosynthesis, as well as plant hormone signaling cascades(**Figure 3C**).

In contrast, the transcriptional response under zinc deficiency exhibited greater specificity, with enriched GO terms largely restricted to phosphate starvation response and oxidant response. (**Figure 3B**). At the pathway level, Zn deficiency was characterized by the upregulation of secondary metabolic pathways, including phenylpropanoid biosynthesis and anthocyanin biosynthesis. (**Figure 3D**). These findings indicate that under Zn-deficient conditions, plants preferentially activate antioxidant defense systems and modulate carbon metabolic flux to mitigate oxidative damage.

### 3.4 Gene expression in the ginsenoside biosynthesis pathway under iron and zinc deficiency

To identify genes involved in the ginsenoside biosynthesis pathway, we compared gene expression patterns between the iron- and zinc-deficiency stress groups and the optimal-concentration control group. In the saponin biosynthesis pathway, 14 genes showed expression changes after stress treatment.

**Figure 4:**
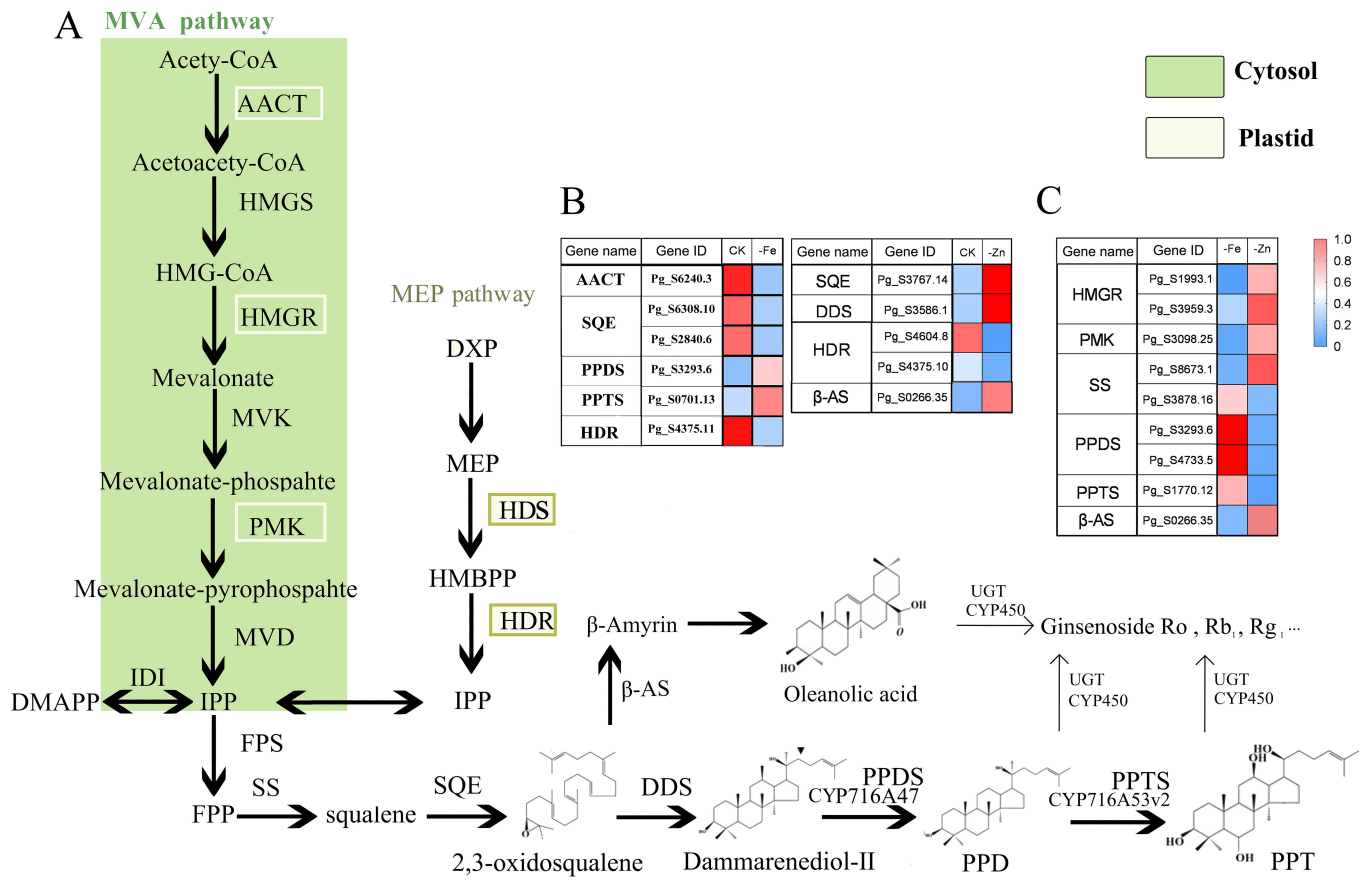
Changes in the ginsenoside biosynthesis pathway and differential gene expression. **(A)** ginsenoside biosynthesis pathway; **(B, C)** Heatmap of gene expression levels. CK, -Fe and-Zn represented control, iron deficiency stress, and zinc deficiency stress, respectively.

Our results showed that the expression levels of several key functional genes were affected, including AACT, HMGR, PMK, SS, SQE, PPDS, PPTS, and the MEP pathway (HDR, HDS) (**Figure 4**, **Supplementary Table S3**). And the changes of multiple UGT and CYP450 families. Under iron deficiency stress, the expression of AACT saponin precursor synthase, as well as the saponin synthesis key enzyme SQE, decreased. Similarly, under zinc deficiency stress, decreased HDR enzyme expression, a core component of the MEP pathway, also affected precursor synthesis. Although SQE and DDS expression levels were slightly higher than in the control, the absence of precursors ultimately reduced saponin content.

Notably, Fe deficiency predominantly influenced the expression of enzymes functioning in the initial stage of ginsenoside biosynthesis. In contrast, Zn deficiency largely impaired the expression of key enzymes in the second and third stages. Notably, β-AS expression was upregulated under Zn deficiency, potentially redirecting flux from 2,3-oxidosqualene toward the saponin Ro biosynthesis branch, which may account for the concurrent reduction in both PPD- and PPT-type ginsenosides. Under iron deficiency stress, the expression levels of PPDS and PPTS in ginseng were significantly higher than those under zinc deficiency stress, and this difference may be the reason for the maintenance of ppt-type ginsenoside content.

### 3.5 Expression of plant hormone-related genes under iron and zinc deficiency

**Figure 5:**
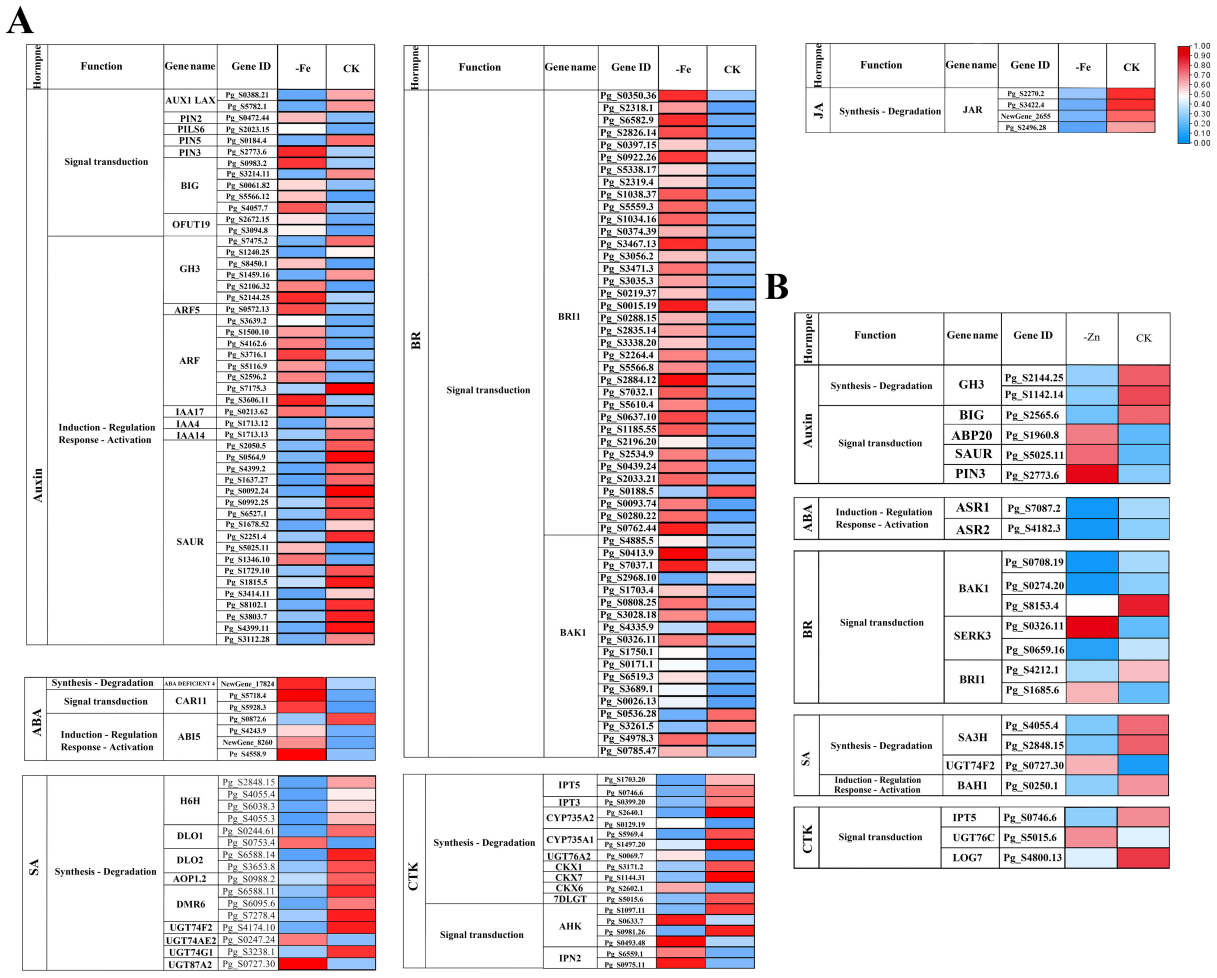
Hormone−related gene expression under -Fe and -Zn. **(A)** Heatmap of differential expression of plant hormone-related genes in ginseng adventitious roots under iron deficiency stress. **(B)** Heatmap of differential expression of plant hormone-related genes in ginseng adventitious roots under zinc deficiency stress. CK, -Fe and-Zn represented control, iron deficiency stress, and zinc deficiency stress, respectively.

Transcriptome analysis revealed that iron and zinc deficiency significantly altered the expression levels of genes involved in hormone signaling pathways in plants. **Figure 5** highlights six hormone pathways with more significant expression changes, including auxin, abscisic acid (ABA), salicylic acid (SA), brassinosteroid (BR), cytokinin (CTK), and jasmonic acid (JA) pathways. **Supplementary Table S4** presents complete information on DEGs for all eight hormones (new gibberellin GA and ethylene pathways).

Under Fe deficiency, genes involved in Signal transduction (e.g., CARI1: Pg_S5718.4, Pg_S5928.3) and Induction–Regulation & Response–Activation (e.g., ABI5: Pg_S0872.6, Pg_S4243.9, NewGene_8260, Pg_S4558.9) within the ABA pathway showed a strong up-regulation trend. In the BR pathway, nearly all identified genes related to Signal transduction (including BRI1, such as Pg_S6582.9 and Pg_S2826.14, and BAK1, such as Pg_S0413.9 and Pg_S7037.1) were upregulated. In contrast, most genes associated with the SA and CTK pathways were down-regulated, whereas genes in the JA pathway were consistently down-regulated. Auxin-related genes displayed a mixed expression pattern. Under Zn deficiency, Induction–Regulation & Response–Activation genes in the ABA pathway (e.g., ASR1: Pg_S7087.2) were down-regulated. In contrast, the BR, SA, CTK, and auxin pathways did not show a consistent directional trend in expression changes, with both up- and down-regulated genes observed across functional categories (Synthesis–Degradation, Signal transduction, and Induction–Regulation & Response–Activation).

### 3.6 Expression of iron and zinc absorption and transport-related genes under iron and zinc deficiency

**Figure 6:**
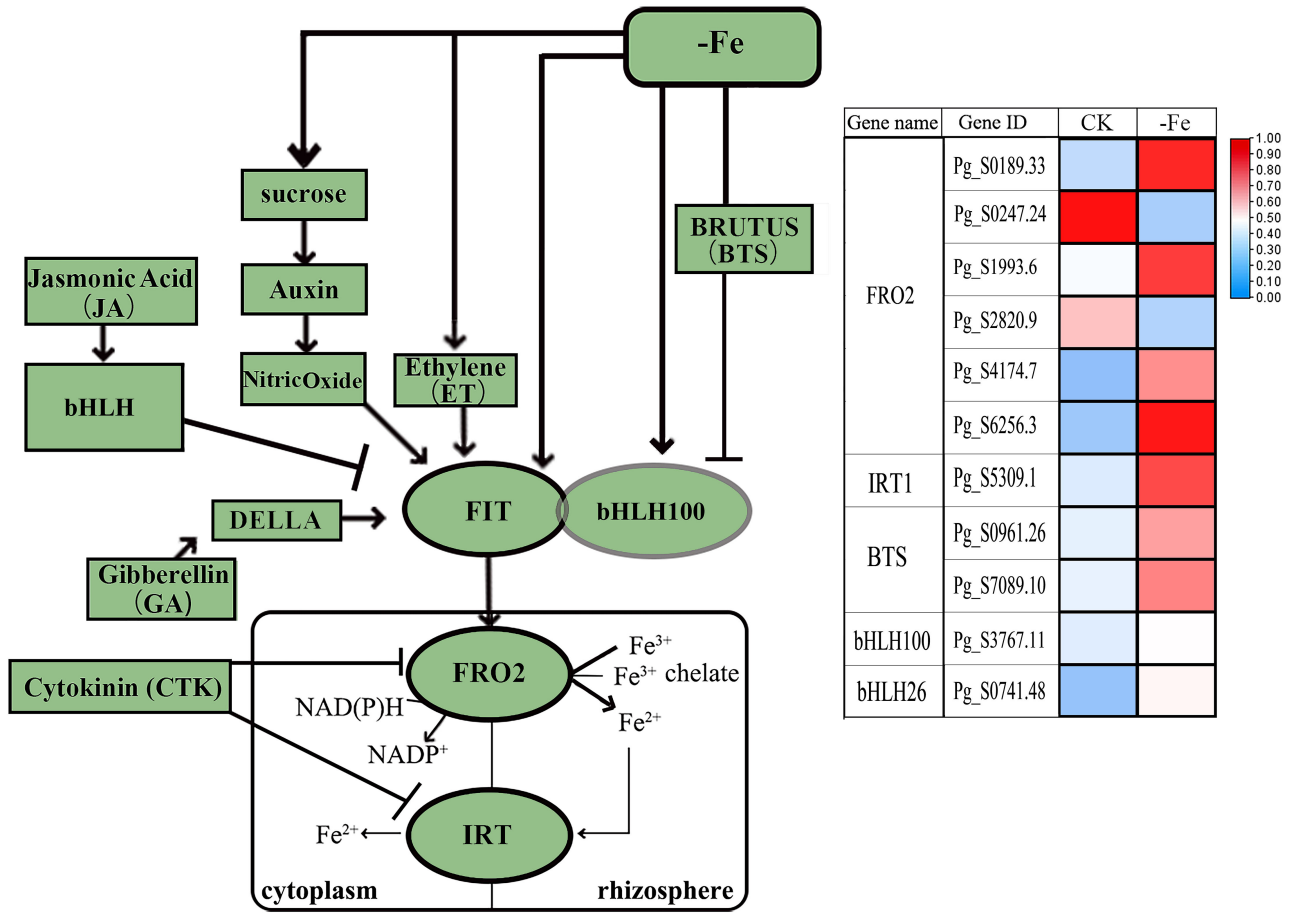
Response to iron deficiency pathway and related gene expression. CK and -Fe represented control and iron deficiency stress, respectively.

**Figure 7:**
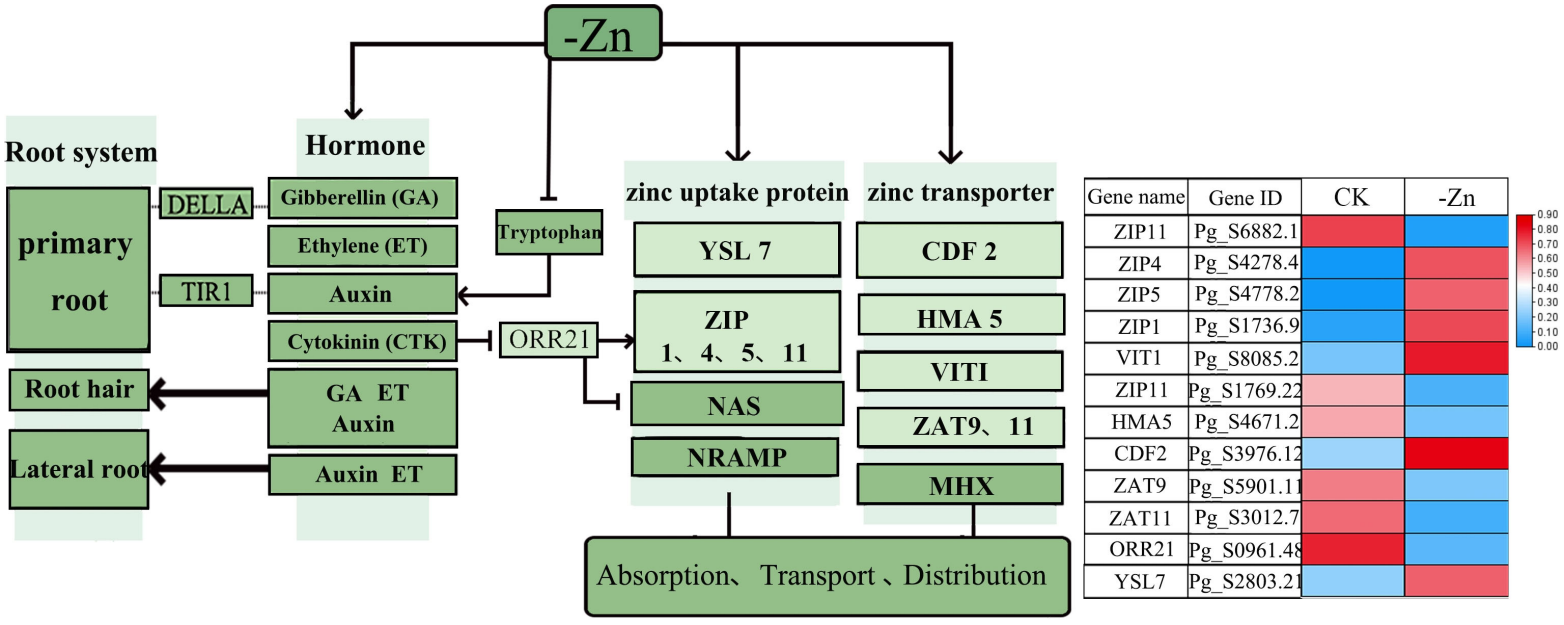
Effects of hormones on roots and the expression of zinc absorption and transport proteins under zinc deficiency. Light green indicates the differential genes in this study, while dark green indicates the genes not differentially expressed but involved in the pathway in this study. CK and -Zn represented control and zinc deficiency stress, respectively.

Transcriptome analysis revealed the core regulatory pathways of ginseng adventitious roots in response to iron and zinc deficiency. In iron deficiency (**Figure 6**, **Supplementary Table S5**), sucrose and hormone signaling pathways were altered, thereby modulating key transcription factors (e.g., FIT, bHLH100) and repressing the negative regulator BRUTUS (BTS). Consequently, the downstream iron-reductase gene FRO2 and the iron-transporter gene *IRT1* were specifically and significantly upregulated. In contrast, zinc deficiency modulated hormone-related pathways involved in root development and triggered a broader differential expression profile of transporter genes. This included the coordinated upregulation of multiple ZIP family genes (e.g., *ZIP1/4/5/11*) and *YSL7*, alongside the downregulation of genes such as *ZAT9/11*. (**Figure 7**, **Supplementary Table S6**).

### 3.7 Validation of transcriptome reliability by qRT-PCR results

**Figure 8:**
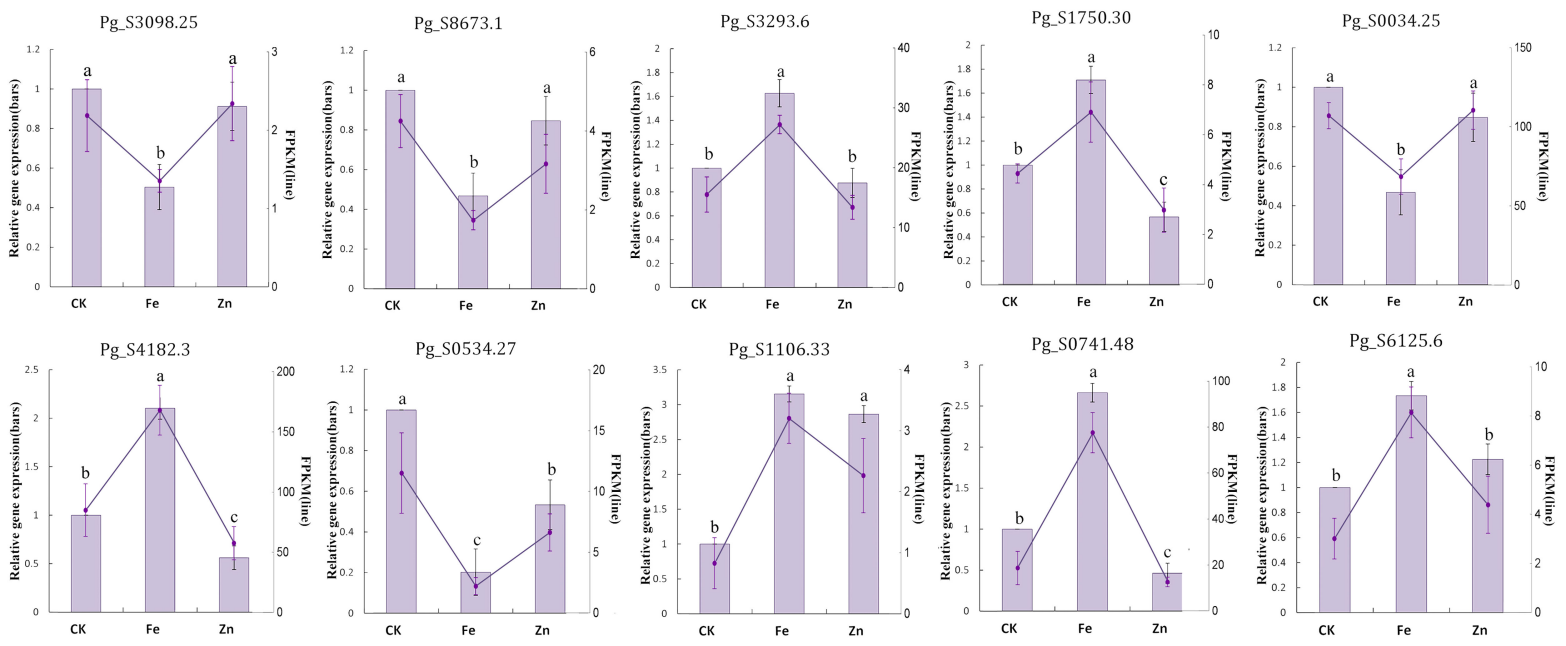
The results of the expression of ten genes amplified by qRT-PCR. Significant differences among groups are denoted by distinct lowercase letters.

To validate the reliability of the transcriptomic data, 10 differentially expressed genes (DEGs) with significant expression changes were selected for qRT-PCR validation. As shown in **Figure 8**, the expression patterns of these genes (Pg_S3098.25, Pg_S8673.1, Pg_S3293.6, Pg_S1750.30, Pg_S0034.25, Pg_S4182.3, Pg_S0534.27, Pg_S1106.33, Pg_S0741.48, and Pg_S6125.6) exhibited high consistency between qRT-PCR and RNA-seq results, thereby confirming the robustness and reliability of the transcriptomic analysis.

## 4 Discussion

**Figure 9:**
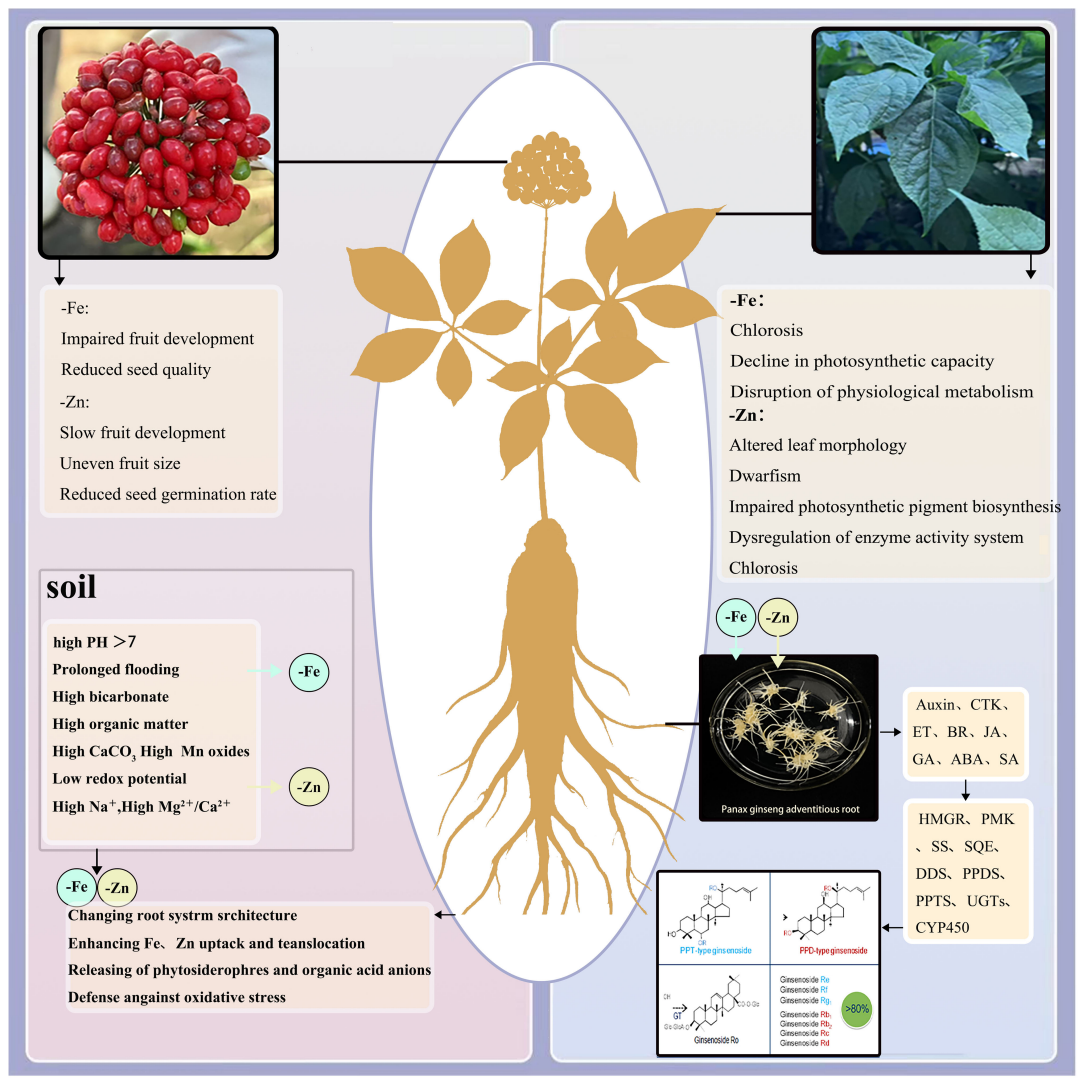
Ginseng’s responses to Iron deficiency and zinc deficiency.

Ginseng is a perennial herb with a slow growth rate, and its root phenotype is affected by genetic, environmental, and physiological factors (Xiang et al., 2022). However, soil-related issues frequently reduce the bioavailability of Fe and Zn, thereby inducing adaptive modifications in plants to cope with stress responses (**Figure 9**). Metal elements are essential for normal plant growth and development (Dubey and Yadav, 2025). It has been reported that, in response to trace element deficiency, iron and zinc, as cofactors for various enzymes, become limiting, thereby impeding cell division and elongation. Iron deficiency directly affects the development of adventitious roots (Hilo et al., 2017). In cucumber, for example, iron deficiency induces distinct morphological alterations, resulting in shortened lateral roots (Pavlovic et al., 2013). In the present study, adventitious roots under control conditions exhibited intact morphology, dense branching, and vigorous elongation. In contrast, Fe deficient treatment significantly reduced root diameter and suppressed lateral root initiation, resulting in a thinner, sparser root architecture. Zinc deficiency is known to exert an even greater adverse impact on plant growth (Bernardy et al., 2016). Studies have reported that one-year-old ginseng plants exhibit stunted root growth as a symptom of zinc deficiency (Ren et al., 2008). In this study, compared to the control group, roots under zinc deficiency exhibited tissue shrinkage and partial necrosis, with overall growth significantly suppressed. The antioxidant system exhibited distinct responses under Fe and Zn deficiency. Fe deprivation reduced the activities of POD, SOD, and CAT, aligning with the previously reported positive correlation between Fe availability and antioxidant capacity in ginseng (Gao et al., 2012; Zhang et al., 2013). In contrast, Zn deficiency increased POD activity but strongly suppressed both SOD and CAT. The observed decline in SOD—a key Zn-dependent enzyme responsible for superoxide radical scavenging and membrane protection—indicates impaired metalloenzyme function under Zn limitation (Qi et al., 2015). This suppression, together with the markedly diminished CAT activity, likely exacerbates oxidative stress and contributes to the accelerated aging phenotype in Zn deficient root tissues.

Both stresses significantly enriched phenylpropanoid biosynthesis and amino acid metabolism pathways. As the core secondary metabolic pathway in plants, phenylpropanoid metabolism produces protective compounds like flavonoids and lignin, which are crucial for stress adaptation. This enrichment suggests that ginseng initiates an early protective response to metal deficiency stress (Zhou et al., 2025). Furthermore, iron deficiency stress was significantly enriched in plant hormone signaling and terpenoid biosynthesis pathways, whereas zinc deficiency stress was enriched in ubiquinone and terpenoid quinone synthesis pathways. These pathways are all related to the biosynthesis of ginsenosides. Integrating the upregulated GO and KEGG results, it is evident that although both iron and zinc deficiency can induce conserved defense-related signaling pathways, their core regulatory mechanisms are fundamentally different: iron deficiency drives a global metabolic reprogramming in plants, characterized by defense features resembling biotic stress responses and extensive remodeling of the lipid system, whereas zinc deficiency activates explicitly the plant’s antioxidant module to maintain intracellular redox homeostasis.

Based on these results, the differentially expressed genes were enriched for the saponin synthesis pathway, with 14 genes identified, including several CYP450 and UGT genes. Saponin biosynthesis was significantly modulated: Fe deficiency downregulated key mevalonate (MVA) pathway genes (e.g., HMGR), reducing precursor supply. Zn deficiency suppressed the methylerythritol phosphate (MEP) pathway gene HDR and altered downstream flux, notably upregulating β-AS, potentially shifting synthesis toward saponin Ro. Consistent with prior studies, the observed “low-upstream, high-downstream” transcriptional shift points to a metabolic trade-off under nutrient stress. To cope with stress, the plant appears to compensate for constrained precursor synthesis by upregulating specific branch-pathway enzymes, potentially favoring defense compounds (e.g., DDS) at the expense of growth (Yang et al., 2025). Therefore, ginsenoside production encounters a fundamental trade-off under nutrient stress: both Fe and Zn deficiencies modulate precursor genes required for saponin synthesis and directly affect yield. Consequently, alongside the inhibition of adventitious root growth, the total saponin content decreased by 31.48% under Fe deficiency and 41.43% under Zn deficiency. Interestingly, the upregulation of PPDS and PPTS under Fe deficiency may explain the stable levels of PPT-type ginsenosides under this specific stress.

The differential expression of hormone-related genes reveals a complex regulatory network underlying the plant stress response (Chen K. et al., 2019). Under iron-deficiency stress, the ABA signaling pathway is activated, whereas the GA, SA, and JA signaling pathways are suppressed. The auxin signaling pathway was activated explicitly under both stresses. In contrast, the ABA pathway exhibited an opposite response pattern (significantly up−regulated under Fe deficiency and down−regulated under Zn deficiency). Other hormonal pathways also exhibited distinct expression patterns between the two stresses, suggesting that hormone-specific signaling reprogramming may contribute to the differential root morphological changes and saponin accumulation under Fe- or Zn-deficiency. Studies have shown that auxin levels are positively correlated with taproot expansion in ginseng (Zhang et al., 2024). The coordinated action of these hormones contributes to the morphological changes observed in ginseng roots under stress. JA is a conserved inducer of plant secondary metabolism and can promote terpenoid biosynthesis, as widely documented in ginseng research. Evidence indicates that JA enhances ginsenoside synthesis (Chen K. et al., 2019). Notably, the JA−signaling gene JAR was down−regulated under Fe deficiency, which may suppress JA−mediated activation of secondary metabolism and potentially exacerbate the loss of saponins (De Geyter et al., 2012).

Transcriptome analysis further elucidated the metabolic pathways and key genes involved in stress response. In Arabidopsis, iron deficiency coordinately induces *FRO2* and *IRT1*, which encode ferric reductase and the iron-regulated transporter, respectively. The bHLH transcription factor network centrally regulates iron uptake and homeostasis in Strategy I plants. Under deficiency, bHLH activates *FRO2* and *IRT1* to enhance Fe³^+^ reduction and Fe²^+^ uptake; it also indirectly regulates vacuolar iron transporter *VIT1* to modulate iron storage. From the perspective of the regulatory process, iron-deficiency signals affect sucrose, auxin, nitric oxide, and ethylene, among others, and also interact with the iron homeostasis-related protein BTS. These signals ultimately converge into a transcriptional complex containing FIT and bHLH factors that regulates the expression of key iron-uptake genes FRO2 and IRT1 (Kobayashi et al., 2007; Long et al., 2010). In ginseng adventitious roots, iron deficiency significantly altered bHLH transcription factor expression, subsequently affecting *FRO2* and *IRT1*, consistent with the conserved Strategy I mechanism (**Figure 6**) (Zhang et al., 2021).

Zinc, the second most abundant transition metal in organisms, serves as an essential cofactor across all six enzyme classes, and its deficiency severely restricts plant growth globally (Broadley et al., 2007; Jin et al., 2014). Homeostasis is maintained by multiple transporter families, including ZIP, HMA, NRAMP, VIT, and YSL (Li et al., 2013; Bozzi and Gaudet, 2021; Stanton et al., 2022), and is closely linked to hormone signaling networks (Zeng et al., 2021). Key phytohormones such as GA, ethylene, auxin, and cytokinin modulate root development through regulators such as DELLA and TIR1, as well as via intermediates including Trp and the transcription factor ORR21, which directly regulates ZIP gene expression (Gupta et al., 2016; Kieber and Schaller, 2018).In this study, zinc deficiency significantly altered the expression of ORR21, ZIP4, and YSL, further supporting the integration of zinc sensing with hormonal and developmental regulation. To elucidate the molecular framework underlying the inhibition of saponin accumulation under Fe/Zn deficiency, we integrated differential expression profiles with known functional genes to develop a preliminary structure–function hypothesis. This model highlights key candidate genes, including saponin biosynthesis genes (*HMGR, PMK, SS*), metal transporters (*IRT1, ZIP4*), and hormone signaling components. These targets provide a clear direction for subsequent functional validation, which will further clarify the molecular mechanisms of ginseng adaptation to nutrient stress.

## 5 Conclusion

In this study, we systematically elucidated the molecular mechanisms underlying the response of Panax ginseng adventitious roots to iron (Fe) and zinc (Zn) deficiencies through integrated physiological and transcriptomic analyses. Our experimental results demonstrated that both Fe and Zn deprivation significantly inhibited root morphological development and induced dynamic changes in antioxidant enzyme activities. These alterations adversely affected the biosynthetic basis of ginsenosides, resulting in a marked reduction in ginsenoside accumulation. Notably, Fe deficiency specifically suppressed the synthesis of protopanaxadiol (PPD)-type ginsenosides, while Zn deficiency resulted in decreased levels of both PPD- and protopanaxtriol (PPT)-type ginsenosides.

Transcriptome analysis revealed that deficiencies in iron and zinc downregulated the expression of enzymes involved in various stages of the saponin biosynthesis pathway, which corresponded with the observed variations in ginsenoside content. Furthermore, dynamic changes in endogenous phytohormones have been implicated in modulating ginsenoside biosynthesis under these nutrient stresses. Collectively, this study provides novel insights into the genetic regulatory mechanisms of Panax ginseng under iron and zinc deficiency, and establishes a theoretical foundation for optimizing bioreactor processes to enable precise control of ginsenoside biosynthesis.

## Data Availability

The original contributions presented in the study are publicly available. This data can be found here: https://www.ncbi.nlm.nih.gov/sra/PRJNA1291973.

## Supplementary material

Supplementary Figure 1 Three stages in saponin biosynthesis pathway.

Supplementary Figure 2 Differentially expressed genes GO annotation classification statistics.

Supplementary Figure 3 Principal component analysis (PCA) of transcriptome profiles.

Supplementary Figure 4 Bubble plot of GO and KEGG enrichment analysis for down-regulated differentially expressed genes.

Supplementary Table 1 List of up-regulated and down-regulated DEGs enriched in biological process GO terms.

Supplementary Table 2 List of up-regulated and down-regulated DEGs enriched in KEGG pathways.

Supplementary Table 3 The data of Ginsenoside Biosynthetic Pathway gene expression analysis.

Supplementary Table 4 The list of DEGs with phytohormone-related genes.

Supplementary Table 5 The list of DEGs with iron deficiency stress genes.

Supplementary Table 6 The list of DEGs with zinc deficiency stress genes.

Supplementary Table 7 Quality control data of RNA-seq.

Supplementary Table 8 Mapped data of clean reads against the reference genome.

Supplementary Table 9 Primers used for qRT-PCR assays.
