# Supplementary figures and images for: Comparative transcriptome and physiological analyses unveil differential responses of Panax ginseng adventitious roots to iron vs. zinc deficiency
Source: Front Plant Sci. 2026 Jan 30;17:1744224. doi: 10.3389/fpls.2026.1744224 (PMC12901382; doi:10.3389/fpls.2026.1744224)

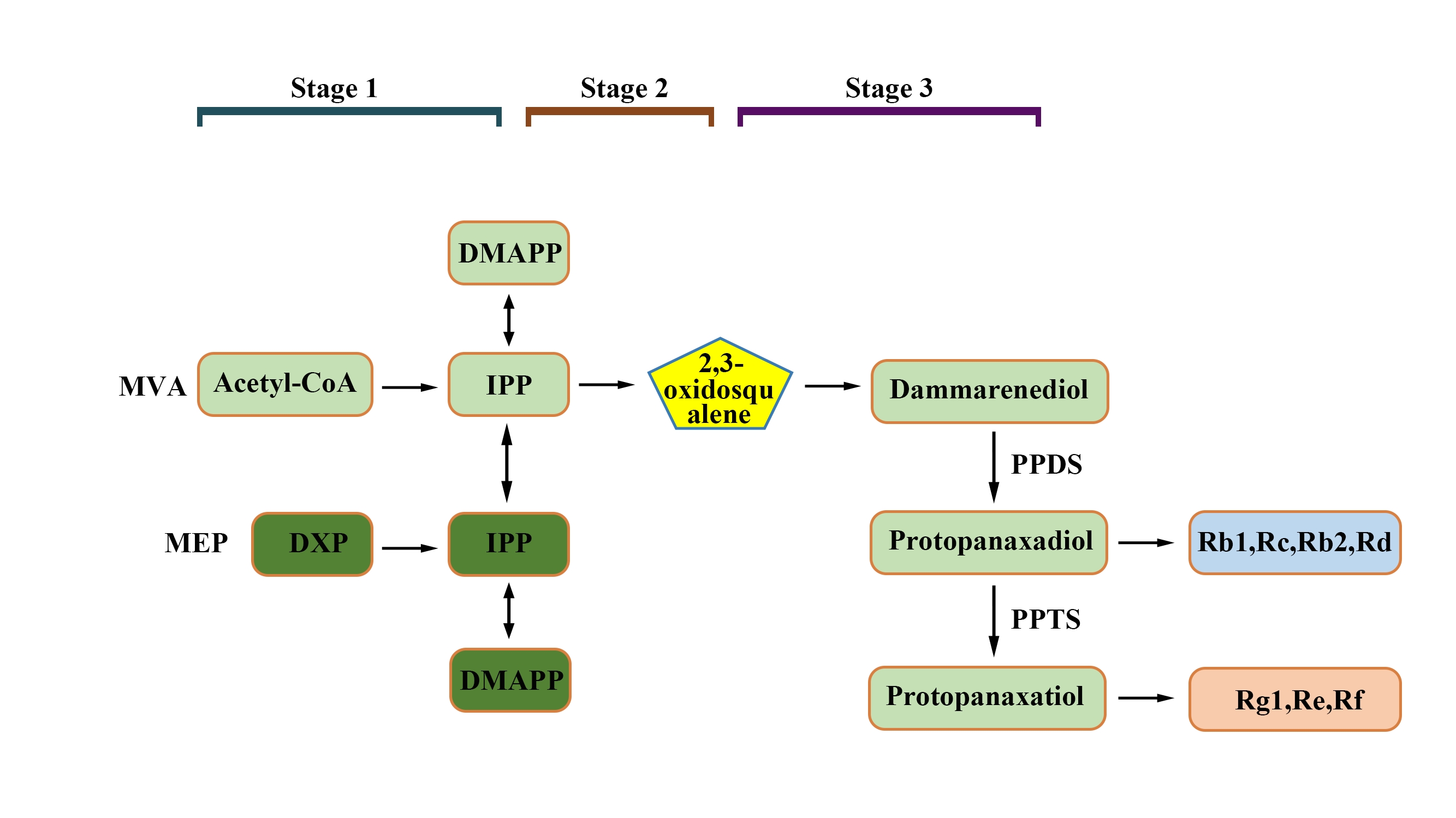

Supplement: Supplementary Figure 1 — Three stages in saponin biosynthesis pathway. [file Image1.jpeg]

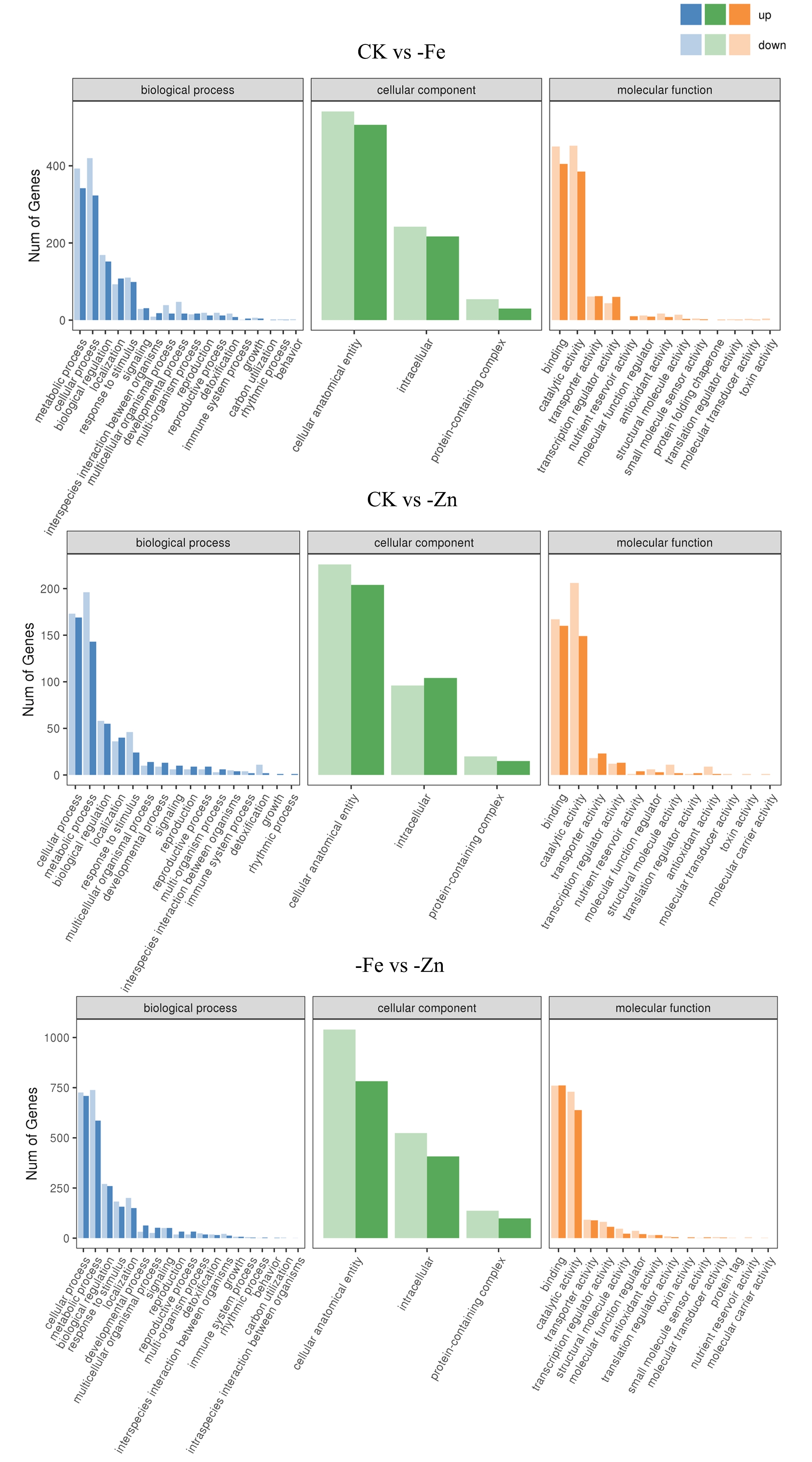

Supplement: Supplementary Figure 2 — Differentially expressed genes GO annotation classification statistics. [file Image2.jpeg]

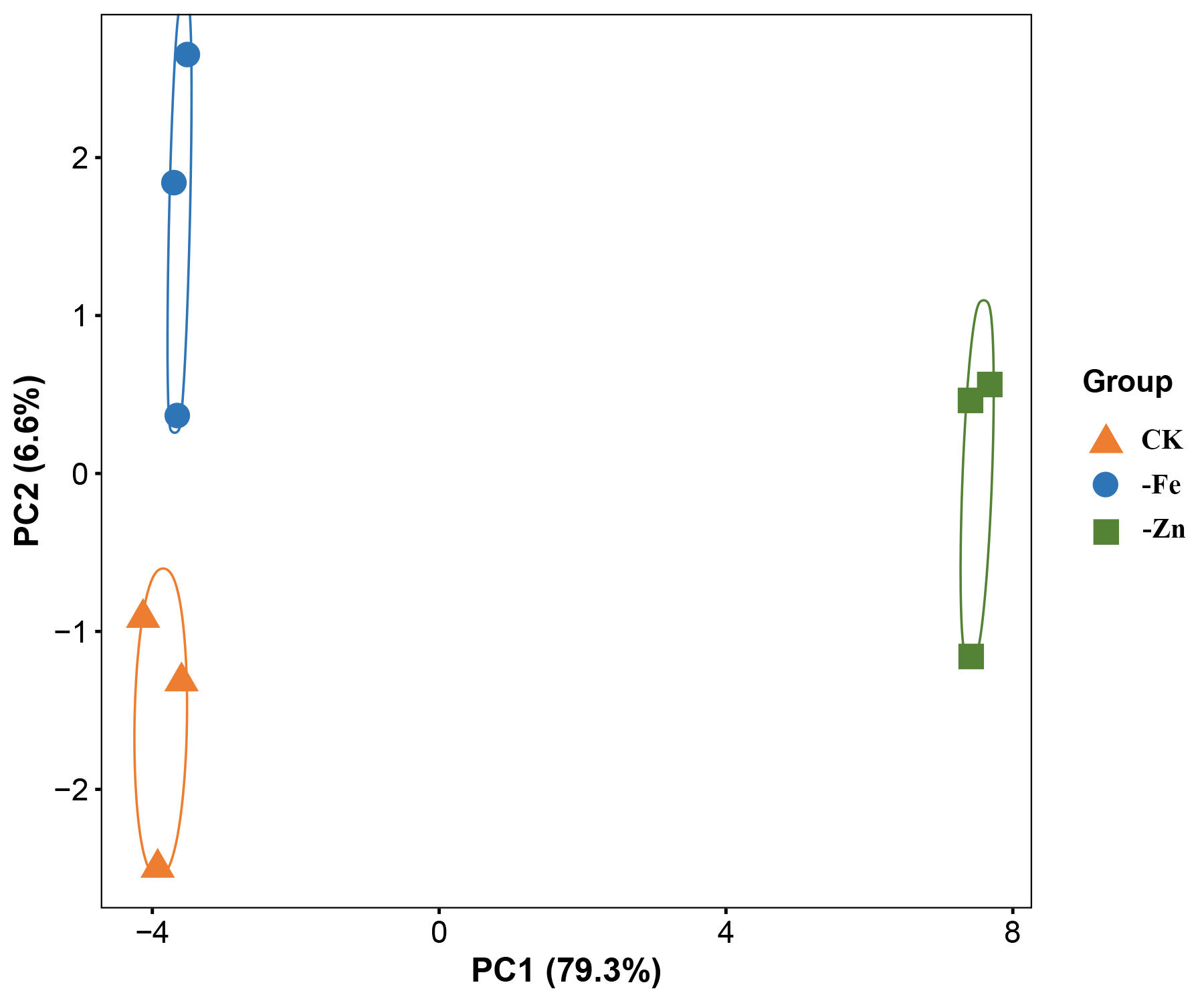

Supplement: Supplementary Figure 3 — Principal component analysis (PCA) of transcriptome profiles. [file Image3.jpeg]
